# Association between Early-Life Self-Medication and Multiple Sclerosis Risk: A Case-Control Study

**DOI:** 10.2174/0118715303412026251125095355

**Published:** 2026-01-26

**Authors:** Seyyed Amin Seyyed Rezaie, Vahid Asgharzadeh, Behroz Mahdavipoor, Mohammad Asgharzadeh, Jalil Rashedi, Bita Alizadeh, Saba Monadi Oskouei, Yassin Siahi, Mahdi Asghari Ozma

**Affiliations:** 1 Student Research Committee, Tabriz University of Medical Sciences, Tabriz, Iran;; 2 Department of Laboratory Sciences, Faculty of Paramedicine, Tabriz University of Medical Sciences, Tabriz, Iran;; 3 Biotechnology Research Center, Tabriz University of Medical Sciences, Tabriz, Iran;; 4 Tuberculosis and Lung Disease Research Center, Tabriz University of Medical Sciences, Tabriz, Iran

**Keywords:** Self-medication, multiple sclerosis, risk factors, early-life exposure, drug use, chronic autoimmune disease, nervous system

## Abstract

**Introduction:**

Multiple sclerosis (MS) is a chronic autoimmune disease of the central nervous system, influenced by both genetic and environmental factors. Among the environmental factors, early childhood self-medication may be relevant in MS development. This study aimed to evaluate the association between MS risk and self-medication practices before the age of 15.

**Methods:**

Under a case-control approach, 260 demographically matched healthy controls from the Azeri community and 469 MS patients completed a comprehensive questionnaire on childhood self-medication history. Regarding the kind and frequency of drug usage, statistical comparisons were made among the groups using the chi-square test.

**Results:**

Self-medication was common in both cohorts; however, individuals with multiple sclerosis exhibited a markedly greater use of corticosteroids, analgesics, and hypnotics (*p* < 0.05). Conversely, healthy controls reported more frequent use of iron and calcium supplements, antihistamines, acetaminophen, antibiotics, and adult cold medications (*p* < 0.05). No significant differences were observed for multivitamins, vitamin D, or non-steroidal anti-inflammatory drugs (*p* > 0.05).

**Discussion:**

This study suggests that unsupervised use of certain medications, such as corticosteroids and hypnotics, before age 15 may increase the risk of developing multiple sclerosis, while common supplements and over-the-counter drugs may have a protective effect. Responsible drug use in childhood should be emphasized through education and regulation.

**Conclusion:**

Early-life use of certain medications, especially corticosteroids and hypnotics, may be linked to increased MS risk, while the use of supplements and common over-the-counter drugs might have protective associations. Educational and regulatory efforts are needed to prevent unsupervised medication use in children.

## 1 INTRODUCTION

Multiple sclerosis (MS) is widely recognized as a chronic inflammatory disorder of the central nervous system (CNS) characterized by inflammatory lesions, demyelinating plaques, and progressive axonal damage [1, 2]. From an epidemiological perspective, MS primarily affects individuals in their 20s and 30s and occurs more frequently in women [3, 4]. In MS, neuroinflammation leads to a wide range of clinical manifestations, such as visual disturbances, motor impairments, mood changes, depression, and anxiety [5]. MS typically presents as relapsing-remitting MS (RRMS), initially manifesting as episodes of neurological symptom worsening followed by partial or complete remission [6, 7]. Over 80% of individuals with RRMS eventually progress to secondary progressive multiple sclerosis (SPMS), which is characterized by a continuous and irreversible decline in neurological function [8].

Although disease-modifying therapies (DMTs) might extend the interval between relapse onset and the onset of secondary progression, no drug with demonstrated efficacy has been identified to prevent the development of SPMS [9]. Although the exact etiology of MS remains unknown, it is widely accepted that the disease arises from a complex interplay of environmental factors and genetic predisposition, which subsequently triggers an autoimmune response against myelin antigens in the central nervous system [10, 11]. Influenced by genetic and environmental factors, activated immune cells, specifically T helper 1 (Th1), T helper 17 (Th17), and B cells, invade the CNS, targeting the myelin sheath and resulting in the development of distinctive plaques in both gray and white matter [12].

**Fig. (1):**
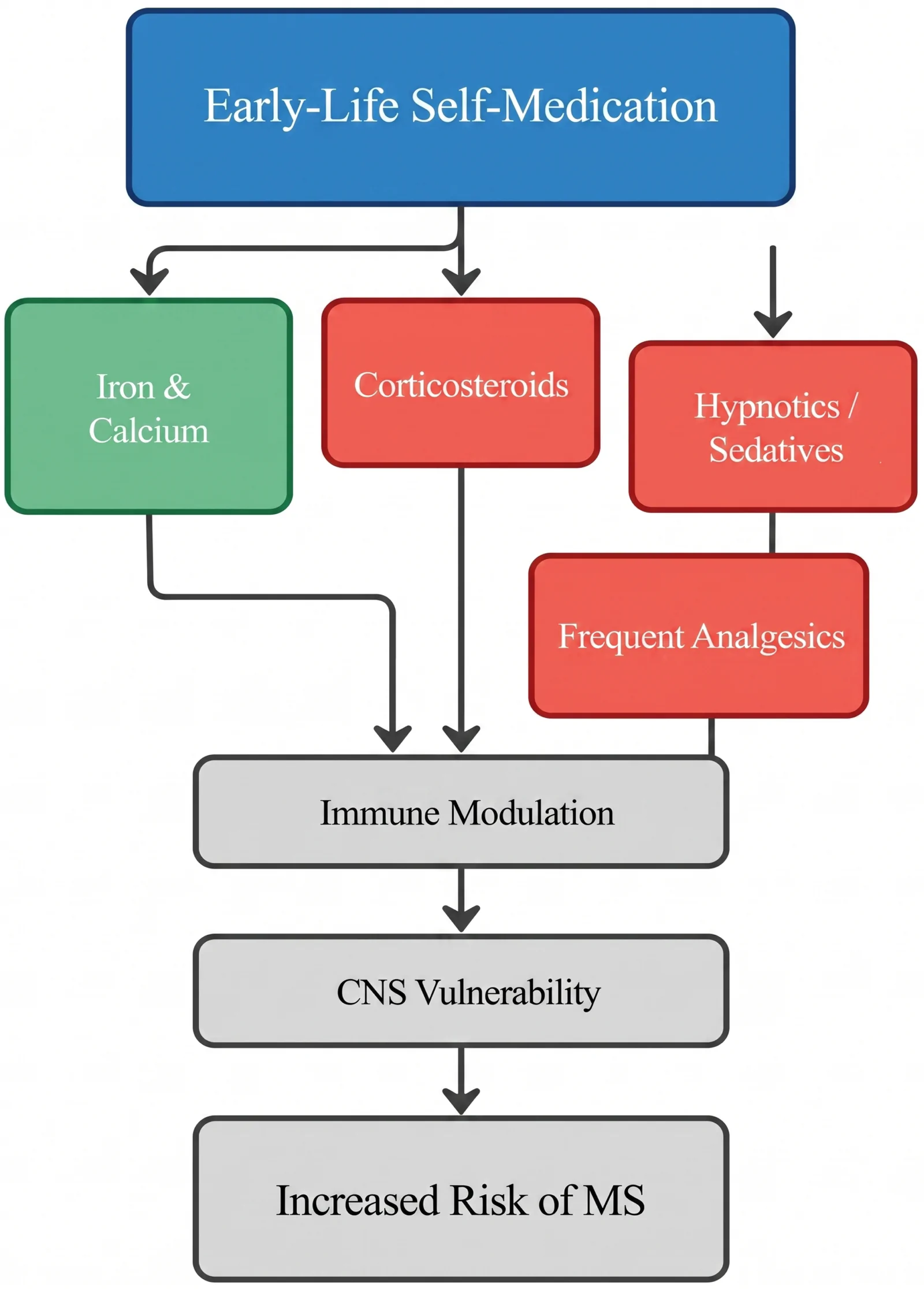
The relationship between early-life self-medication and increased risk of developing MS.

Genetic variables linked to MS include polymorphisms in the vitamin D receptor, interleukin-10 promoter, and TNF-α gene [13]. Nevertheless, these genetic traits are not easily altered, and current methods are challenging to manipulate across many populations [14]. On the other hand, addressing environmental factors is more practical, and interventions targeting them can be implemented across all societies [15]. Significant environmental influences include diet, Epstein–Barr virus (EBV) exposure, and tobacco use (Fig. **1**) [16, 17].

More research is needed to understand how various environmental factors, particularly the indiscriminate use of medications, may influence the likelihood of developing MS or modifying its risk. Further investigation into the relationship between environmental exposures, including self-administered drug use, and multiple sclerosis will help clarify the underlying mechanisms of the disease and guide more targeted prevention strategies. To increase awareness of the risks associated with self-medication and its potential impact on MS, the present study examined the association between self-medication, defined as the use of drugs without a prescription or medical supervision, and MS in the Azeri population of Iran.

## 2 MATERIALS AND METHODS

### 2.1 Study Design and Population

This was a case-control study conducted among the Azeri community living in East Azerbaijan Province, Iran. The research sample included 469 patients diagnosed with MS and 260 unrelated healthy controls. The diagnosis of MS was confirmed by neurologist investigators using clinical and paraclinical criteria, including MRI findings and cerebrospinal fluid (CSF) analysis, in accordance with the McDonald criteria [18]. Control subjects were selected based on the absence of neurological, autoimmune, and inflammatory disorders, as well as no family history of MS in their first-degree relatives. The study protocol was approved by the Ethics Committee of Tabriz University of Medical Sciences (IR.TBZMED.REC.1397.889).

### 2.2 Inclusion and Exclusion Criteria

The inclusion criteria for MS patients required a confirmed diagnosis of MS based on established clinical and paraclinical evaluations. All patients were of Azeri ethnicity and provided written informed consent to participate in the study. Healthy control participants were also of Azeri ethnicity. They were selected based on the absence of neurological, autoimmune, or inflammatory disorders and no family history of MS among their first-degree relatives. All controls voluntarily agreed to take part in the study. Participants who submitted incomplete or internally inconsistent responses to the questionnaire were excluded. Contradictory responses were defined as logically incompatible answers to related items (*e.g.*, reporting both frequent and never use of the same drug). Additionally, individuals with a documented history of chronic systemic illnesses that could potentially confound the relationship between self-medication and MS risk were also excluded from the final analysis.

### 2.3 Data Collection

Data collection was performed using a standardized, self-administered questionnaire specifically designed to assess participants’ history of unsupervised medication use before the age of 15. The questionnaire included detailed items on the types of medications used, the frequency and duration of use, and the reasons for engaging in self-medication. Participants completed the questionnaire independently, with research personnel available to provide clarification when needed to ensure accuracy and completeness. To minimize recall bias, examples of commonly used childhood medications were provided to help facilitate participants’ recollection.

### 2.4 Definition of Self-Medication

This study defined self-medication as the use of pharmaceutical products without a prescription or direct supervision by a qualified healthcare professional, specifically before participants reached 15 years of age [19]. This definition encompasses both prescription medications and over-the-counter (OTC) drugs, including analgesics, cold remedies, supplements, and antibiotics, which are commonly available without medical authorization. Medications were categorized by pharmacological properties, and the study evaluated the association between different classes of self-administered drugs and the subsequent risk of developing multiple sclerosis.

### 2.5 Ethical Considerations

The research adhered to the principles of the Declaration of Helsinki [20]. Written informed permission was obtained from all participants before participation. Participant confidentiality was rigorously maintained throughout the study.

### 2.6 Statistical Analysis

Statistical analyses were performed using SPSS software (version 26). The primary comparisons between MS patients and healthy controls were conducted using the chi-square test. Although multivariate logistic regression was considered to control for potential confounders, such as age and sex, limitations in available covariate data restricted the analysis. Therefore, only univariate results are presented. A *p*- value of less than 0.05 was considered statistically significant.

## 3 RESULTS

### 3.1 General Prevalence of Self-Medication

A significant fraction of individuals in both the MS patient group and the healthy control group reported self-medicating before the age of 15. Among the 469 MS patients, 55.7% (95% CI: 51.2–60.2; n = 261) reported self-medication, while 52.7% (95% CI: 46.6–58.8; n = 137) of the 260 healthy controls reported the same behavior. The chi-square test (*p* = 0.442) indicated no statistically significant difference between the two groups, suggesting a similarly high prevalence of self-medication during early adolescence in both populations.

### 3.2 Increased Use of Corticosteroids, Hypnotics, and Analgesics in MS Patients

MS patients demonstrated a significantly higher prevalence of self-medication with specific drug classes during childhood compared to healthy controls. Notably, 32.6% of MS patients (n = 153) reported unsupervised use of corticosteroids before the age of 15, in contrast to 2.9% of healthy controls (n = 4) (*p* = 0.001). Similarly, the use of hypnotics was more common among MS patients, with 8.0% (n = 38) reporting use, compared to 1.5% (n = 4) in the control group (*p* = 0.007). In addition, 18.8% of MS patients (n = 88) had self-medicated with analgesics, whereas only 4.4% of healthy controls (n = 6) reported such use (*p* = 0.001).

### 3.3 Higher Consumption of Calcium, Iron, Antihistamines, Antibiotics, Acetaminophen, and Adult Cold Medicines in Healthy Controls

Self-medication with certain over-the-counter drugs and supplements was significantly more common in healthy individuals than in MS patients. Calcium supplementation was reported in 10.8% of healthy controls, compared with 4.2% of MS patients (*p* = 0.01). Similarly, iron supplement usage was higher in healthy individuals (8.8%) than in MS patients (0.8%) (*p* = 0.001). The use of antihistamines was reported by 4.4% of healthy controls, while none of the MS patients reported their use (*p* = 0.001). Antibiotic use was more frequent among healthy controls (5.1%) compared to MS patients (1.5%) (*p* = 0.039). Acetaminophen consumption was significantly greater among healthy individuals (7.3%) than MS patients (0.4%) (*p* = 0.001). Finally, the use of adult cold medications was higher in healthy controls (10.2%) versus MS patients (1.9%) (*p* = 0.001). This higher use in controls may reflect more proactive parental health behaviors, greater use of over-the-counter medications for minor illnesses, or better nutritional support during childhood.

### 3.4 No Significant Differences in the Use of Omeprazole, Tramadol, Diclofenac, Mefenamic Acid, Multivitamins, Vitamin D, and Ibuprofen

Comparative analysis revealed no statistically significant differences between MS patients and healthy controls in the self-reported use of several medications before the age of 15. Specifically, usage rates of omeprazole, tramadol, diclofenac, mefenamic acid, multivitamins, vitamin D, and ibuprofen were similar across both groups (*p* > 0.05 for all comparisons). For example, multivitamin use was reported by 20.4% of healthy individuals and 19.5% of MS patients (*p* = 0.831). Vitamin D use showed a borderline *p*-value (8.8% in controls *vs*. 4.2% in MS patients; *p* = 0.065), suggesting a potential trend toward significance that could become clearer with a larger sample size. Likewise, no significant group differences were observed for the use of nonsteroidal anti-inflammatory drugs (NSAIDs), including diclofenac, ibuprofen, and mefenamic acid, or omeprazole (Table **1**).

## 4 DISCUSSION

Several lifestyle factors influence MS risk. Certain behaviors and exposures may reduce susceptibility, whereas others, particularly those that affect immune regulation during critical developmental periods, may increase the likelihood of disease onset [21]. Given the rising trend of self- medication in Iran and the increasing incidence of MS, this study focused on the Azeri population to investigate the potential association between early-life unsupervised drug use and MS development. Notably, the association between corticosteroid self-medication and MS risk was particularly significant; 32.6% of MS patients reported using corticosteroids without medical supervision before age 15, compared with only 2.9% of healthy controls (*p* = 0.001). Although corticosteroids are widely used for their broad-spectrum anti-inflammatory and immunosuppressive effects, improper or prolonged use, especially during developmental stages, may disrupt immune homeostasis and predispose individuals to autoimmune disorders, such as MS [22, 23].

Several mechanisms support the biological plausibility of this association. A common adverse effect of corticosteroid use is weight gain and increased BMI, both of which have been linked to a higher risk of MS [10]. Corticosteroids may also contribute to sleep disturbances, including insomnia, which further increases MS risk [24]. In addition, glucocorticoids can exert paradoxical effects on the immune system. Specifically, they may enhance the differentiation of T-helper 17 (Th17) cells and promote the release of IL-17, a pro-inflammatory cytokine frequently elevated in the blood and cerebrospinal fluid of MS patients. These observations suggest that early, unsupervised exposure to corticosteroids may induce immune dysregulation and neuroinflammation, thereby increasing the risk of MS later in life [25, 26].

This study identified a statistically significant association between the use of sleeping medications in childhood and the chance of developing multiple sclerosis. It was found that 8% of multiple sclerosis patients used hypnotics without medical supervision before the age of 15, in contrast to 1.5% of healthy controls (*p* = 0.007). These data suggest that early exposure to hypnotic medications may induce multiple sclerosis. Braley *et al.* identified a comparable correlation between the use of sleeping tablets and multiple sclerosis in individuals with the condition [27, 28]. Many individuals who use hypnotic medications experience sleep disturbances, which may help explain the observed association. These drugs are commonly prescribed for insomnia, a condition that has been linked to an increased risk of MS. Chronic sleep disruption can impair immune regulation, elevate systemic inflammation, and alter neuroendocrine signaling, potentially contributing to autoimmunity and neuroinflammatory disorders. Therefore, both the use of hypnotic drugs and the underlying sleep problems they are intended to treat may increase MS risk during early developmental stages [29].

Calcium intake was associated with a significantly reduced risk of MS in our study; 4.2% of patients reported calcium use compared with 10.8% of healthy controls (*p* = 0.01). Similar findings were reported by Abbasi *et al.* in a cohort of 660 MS patients and 421 healthy individuals [30]. In contrast, a prospective study by Cortese *et al.* found no significant association between calcium intake and MS incidence [31]. Differences in genetic backgrounds or environmental factors influencing calcium metabolism across populations may explain these inconsistent results. Calcium, along with magnesium, is essential for the development, structure, and stability of myelin [32]. Insufficient calcium intake during central nervous system development can lead to myelin instability, potentially contributing to demyelination and MS-related symptoms later in life. Therefore, adequate calcium supplementation during childhood may reduce MS risk by supporting proper myelin formation and stability [33].

Iron consumption was also associated with a significantly reduced risk of MS in our study. Only 0.8% of individuals with MS reported iron supplementation compared with 8.8% of healthy controls (*p* = 0.001). Rezaeimanesh *et al.* similarly reported a protective association between iron intake and MS risk, showing that iron-containing dietary supplements reduced the incidence of primary progressive MS (PPMS) in a cohort of 143 patients and 400 controls [34]. Iron is essential for several enzymes involved in myelin synthesis, including fatty acid synthase and other myelin-forming pathways. Therefore, iron deficiency may impair myelin production and increase susceptibility to MS. Conversely, adequate iron intake supports proper myelin synthesis, potentially lowering MS risk [35].

Anemia results from iron deficiency. In multiple sclerosis patients, anemia is often attributed to either iron deficiency or anemia of chronic disease, both of which are linked to an increased risk of MS. Improving iron intake can prevent iron deficiency anemia and, consequently, reduce MS risk [36]. MS occurs more frequently in women than in men, and women generally have lower iron levels. This difference may partly explain the higher prevalence of MS among women [37, 38]. Adequate iron intake may help lower MS risk through multiple mechanisms; however, excessive intake can lead to brain iron accumulation [39]. Abnormal brain iron buildup may trigger lipid peroxidation and neuronal cell death, contributing to neurodegeneration and MS pathogenesis. Therefore, recommended iron intake for MS prevention should be balanced and carefully regulated [40].

We observed that antihistamine use may be associated with a reduced risk of MS. In our study, 4% of healthy participants reported antihistamine use, whereas none of the MS patients did (*p* = 0.001). This finding is consistent with the study by Alonso *et al.*, which included 163 MS patients and ten controls per patient, and found an association between H1 receptor blocker use and reduced MS risk [41]. Histamine is an inflammatory mediator with multiple physiological functions, but excessive histamine activity can be detrimental [42]. It can increase blood–brain barrier permeability, promoting central nervous system inflammation, which may contribute to MS development. Therefore, antihistamines may lower MS risk by mitigating these adverse effects [43]. Supporting this, cerebrospinal fluid histamine levels are significantly higher in MS patients compared with healthy controls matched for age and sex, suggesting a critical role for histamine in the pathogenesis and progression of MS [44]. By blocking histamine activity, antihistamines may thus help prevent or ameliorate MS [45].

Our study found that acetaminophen use was associated with a reduced risk of MS. Only 0.4% of MS patients reported acetaminophen use, compared with 7.3% of healthy individuals (*p* = 0.001). Acetaminophen (APAP) is a non-opioid analgesic with antipyretic effects; it is not classically an NSAID but exerts weak anti-inflammatory activity *via* modulation of cyclooxygenase (COX) pathways [46]. Notably, acetaminophen preferentially inhibits CNS COX pathways with minimal peripheral effects, thereby reducing prostaglandin synthesis, inflammation, and immune activation [47]. By modulating the immune system and lowering prostaglandin levels, acetaminophen may help prevent or mitigate MS [48]. Additionally, acetaminophen can induce leukopenia and neutropenia, potentially reducing CNS leukocyte levels and further lowering MS risk [49].

The study results indicated an inverse association between the use of adult cold medications and MS risk. Use of these medications was significantly lower among MS patients compared with healthy controls (1.9% *vs*. 10.2%, *p* < 0.001). Adult cold remedies are used to alleviate common cold symptoms and may contain varying ingredients depending on the manufacturer. While some components are common to most formulations, others are specific to certain brands [50]. Common ingredients include acetaminophen and either phenylephrine or diphenhydramine [51], whereas guaifenesin is present only in selected formulations [52]. Based on the mechanisms described for acetaminophen, this component may contribute to a reduced MS risk. Additionally, first-generation H1 receptor blockers, such as diphenhydramine, inhibit H1 histamine receptor activation, counteracting histamine’s effects and potentially lowering MS risk [53].

This study found that the use of analgesics, a class of pain-relieving medications, was associated with an increased risk of developing MS [54]. Among participants, 18.8% of MS patients reported using non-acetaminophen analgesics before the age of 15, compared with only 4.4% of healthy controls, a statistically significant difference (*p* = 0.001). Unlike acetaminophen, other analgesics that inhibit the lipoxygenase pathway and generate algogenic metabolites may elevate MS risk [48]. These metabolites, including histamine, glutamate, and prostaglandins, can increase CNS inflammation and contribute to MS development; histamine promotes CNS inflammation [55], glutamate contributes to autoimmune demyelination [56], and prostaglandins drive neuroinflammation and neurodegeneration [57].

The study also found that MS patients reported lower antibiotic use than healthy individuals (*p* < 0.05). While self-medication with antibiotics is discouraged due to serious health risks [58, 59], certain bacteria, such as *Clostridium perfringens*, may contribute to MS pathogenesis, and antibiotics could reduce MS risk by targeting these microbes [60]. However, inappropriate antibiotic use can lead to antibiotic resistance, reducing efficacy and increasing morbidity and mortality from infections [61], as well as altering gut microbiota composition, which may have additional health consequences [62].

Vitamin D supplementation was not associated with reduced MS risk in this study (*p* = 0.065). The lack of significance may be due to the limited sample size and generally low vitamin D intake in both groups. Vitamin D may influence MS risk by modulating lymphocyte activation, T-helper cell differentiation, and immune responses [63]. Given the small study population and low vitamin D consumption, further investigation is warranted to clarify the relationship between vitamin D intake and MS risk in this community.

Pharmaceutical misuse poses a significant public health threat in many communities [64]. Factors contributing to indiscriminate self-medication include the perception that it is harmless, lack of time or access to physicians, pharmacies dispensing drugs without prescriptions, rising medication and treatment costs, and economic rationalization of self- medication [65, 66]. Insufficient knowledge of drug pharmacology, interactions, and potential side effects further exacerbates misuse, posing risks to both individuals and healthcare systems [67]. Therefore, governments should implement evidence-based measures to mitigate this behavior. Potential interventions include incorporating appropriate education on medication use in schools, enforcing stricter regulations on drug marketing and non-prescription sales, and providing universal health coverage to eliminate direct physician fees [68-70].

This study had several limitations. Comprehensive data on participants’ comorbidities were not collected, which may influence both self-medication behaviors and MS risk, potentially serving as confounding factors. Additionally, genetic predisposition to MS was not assessed, limiting the ability to account for hereditary risk differences. The absence of population-specific genetic comparisons constrains interpretation, and future research should incorporate genetic data to more accurately elucidate these relationships.

## CONCLUSION

Self-medication during early life with corticosteroids, hypnotics, and analgesics among multiple sclerosis patients was more prevalent than in healthy controls. Conversely, the control group exhibited greater usage of antihistamines, calcium and iron supplements, antibiotics, acetaminophen, and adult cold medications. These findings suggest that while certain pharmacological agents may possess potential preventive effects, unsupervised administration of these substances in children may correlate with an increased risk of developing multiple sclerosis. Self-medication can negatively impact immune system development and increase susceptibility to autoimmune disorders, as it often involves inappropriate dosages, incorrect timing, and disregard for individual drug sensitivities. Public health initiatives should focus on educating caregivers and implementing regulations to mitigate improper medication use in children.

## Figures and Tables

**Table 1:** Comparison of patients with multiple sclerosis (MS) and healthy individuals in terms of taking medicine without a doctor's prescription.

*P*-Value	Healthy Controls Frequency (%)	MS Patient’s Frequency (%)	-
0.442	137(52.7)	261(55.7)	Taking medicine without a doctor's prescription
0.001	4(2.9)	85(32.6)	Cortone
0.007	2(1.5)	21(8)	Hypnotics
0.831	28(20.4)	51(19.5)	Multivitamins
0.065	12(8.8)	11(4.2)	Vitamin D
0.238	2(1.5)	1(0.4)	Vitamin C
0.01	15(10.8)	11(4.2)	Calcium
0.001	12(8.8)	2(0.8)	Iron
0.039	7(5.1)	4(1.5)	Antibiotics
0.001	6(4.4)	0(0)	Antihistamines
0.238	2(1.5)	1(0.4)	Omeprazole
0.468	0(0)	1(0.4)	Tramadol
0.001	14(10.2)	5(1.9)	Adult cold
0.221	13(9.5)	16(6.1)	Ibuprofen
0.238	2(1.5)	1(0.4)	Mefenamic acid
0.001	10(7.3)	1(0.4)	Acetaminophen
0.238	2(1.5)	1(0.4)	Diclofenac
0.001	6(4.3)	49(18.8)	Other Analgesics
-	260	469	Total

## Data Availability

All data generated or analyzed during this study are included in this published article.

## LIST OF ABBREVIATIONS

MS: Multiple Sclerosis
CNS: Central Nervous System
RRMS: Relapsing-Remitting Multiple Sclerosis
SPMS: Secondary Progressive Multiple Sclerosis
DMTs: Disease-Modifying Treatments
Th1: T Helper 1
Th17: T Helper 17
EBV: Epstein–Barr Virus
MRI: Magnetic Resonance Imaging
CSF: Cerebrospinal Fluid
NSAIDs: Nonsteroidal Anti-Inflammatory Drugs
APAP: Acetaminophen (Paracetamol)
COX: Cyclooxygenase
H1: Histamine Type 1 Receptor

## STANDARDS OF REPORTING

STROBE guidelines were followed.
